# GqPCR-stimulated dephosphorylation of AKT is induced by an IGBP1-mediated PP2A switch

**DOI:** 10.1186/s12964-021-00805-z

**Published:** 2022-01-08

**Authors:** Guy Nadel, Zhong Yao, Ehud Wainstein, Izel Cohen, Ido Ben-Ami, Amir Schajnovitz, Galia Maik-Rachline, Zvi Naor, Benjamin A. Horwitz, Rony Seger

**Affiliations:** 1 Departments of Biological Regulation, The Weizmann Institute of Science, Rehovot, Israel; 2 Present Address: IVF and Fertility Unit, Department of OB/GYN, Shaare Zedek Medical Center and The Hebrew University Medical School, Jerusalem, Israel; 3 Department of Biochemistry and Molecular Biology, Tel Aviv University, Tel Aviv, Israel; 4 Faculty of Biology, Technion–Israel Institute of Technology, Haifa, Israel

**Keywords:** AKT, PI3K, PP2A, IGBP1, PKC

## Abstract

**Background:**

G protein-coupled receptors (GPCRs) usually regulate cellular processes via activation of intracellular signaling pathways. However, we have previously shown that in several cell lines, GqPCRs induce immediate inactivation of the AKT pathway, which leads to JNK-dependent apoptosis. This apoptosis-inducing AKT inactivation is essential for physiological functions of several GqPCRs, including those for PGF2α and GnRH.

**Methods:**

Here we used kinase activity assays of PI3K and followed phosphorylation state of proteins using specific antibodies. In addition, we used coimmunoprecipitation and proximity ligation assays to follow protein–protein interactions. Apoptosis was detected by TUNEL assay and PARP1 cleavage.

**Results:**

We identified the mechanism that allows the unique stimulated inactivation of AKT and show that the main regulator of this process is the phosphatase PP2A, operating with the non-canonical regulatory subunit IGBP1. In resting cells, an IGBP1-PP2Ac dimer binds to PI3K, dephosphorylates the inhibitory pSer608-p85 of PI3K and thus maintains its high basal activity. Upon GqPCR activation, the PP2Ac-IGBP1 dimer detaches from PI3K and thus allows the inhibitory dephosphorylation. At this stage, the free PP2Ac together with IGBP1 and PP2Aa binds to AKT, causing its dephosphorylation and inactivation.

**Conclusion:**

Our results show a stimulated shift of PP2Ac from PI3K to AKT termed “PP2A switch” that represses the PI3K/AKT pathway, providing a unique mechanism of GPCR-stimulated dephosphorylation.

**Video Abstract**

**Supplementary Information:**

The online version contains supplementary material available at 10.1186/s12964-021-00805-z.

## Background

The PI3K/AKT is a central signaling pathway that transmits a variety of extracellular signals to induce cellular processes such as proliferation, survival, metabolism, differentiation, and upon dysregulation also cancer [1–3]. The mechanism by which the PI3K/AKT pathway is activated has been extensively studied. It was shown that the activation involves the recruitment of PI3K to the plasma membrane [4], where it converts the membranal phosphoinositide PIP2 to PIP3 that recruits AKT Ser/Thr kinase to its vicinity. This allows the phosphorylation of Thr308 and Ser473 and binding to membranes that are necessary for the full activation of AKT [5]. Thereafter, activated AKT phosphorylates downstream substrates that regulate transcription, survival, translation, migration and metabolism. As in most signaling pathways, the activation of the PI3K/AKT pathway is transient, and its negative regulators include phospholipid phosphatases (PTEN) at the PI3K level and the protein phosphatases PP2A and PHLPP at the AKT level [6–8].

PP2A is a Ser/Thr protein phosphatase that plays a role in the regulation of many cellular processes [9, 10]. Under most circumstances it acts as a heterotrimer composed of a scaffold (A subunit, PP2Aa), a catalytic (C subunit, PP2Ac) and a regulatory subunit ((B subunit) [11, 12]). While the A and C subunits are composed of two very similar isoforms each, the B subunit consists of up to 17 different proteins, which determine PP2A’s specificity. In addition to the classical heterotrimer, PP2A can act as heterodimers of PP2Ac and PP2Aa subunits [9, 13], or PP2Ac and B subunits [14]. In addition, PP2Ac interacts with another protein termed immunoglobulin-α-binding protein 1 (IGBP1; also known as α4; [15, 16]), to form an active dimer [17, 18]. The ability of PP2Aa to interact with this dimer and affect its activity is still controversial ([15, 19] vs [20, 21]), and needs further clarification.

Importantly, the various PP2A complexes dictate the specificity of the phosphatase to act on processes such as cell cycle progression, metabolism and survival [11, 12]. Thus, PP2A can induce apoptosis in some systems [22], and was also identified as a tumor suppressor protein complex [23, 24]. This activity was shown in mouse models [11], and aberrant expression, mutations and regulation were found in various human malignancies. Importantly, some of the effects of PP2A in survival and apoptosis are due to regulation of the survival signaling protein kinase AKT [25, 26]. Indeed, PP2A dephosphorylates both activatory p-Ser473 and p-Thr308 of AKT, to induce its full inactivation [25]. The AKT-inactivating PP2A in mammals was shown to be composed of the B regulatory PR56β and PR56γ [27], as well as B55 [28, 29] that determine the proper and timely inactivation of AKT.

G protein coupled receptors (GPCRs) are the largest group of membranal proteins that mediate cellular responses to a wide variety of extracellular agents [30–32]. GPCRs function via heterotrimeric G proteins, as well as G-protein independent mechanisms [33, 34], that transmit signals to signaling pathways such as the PI3K/AKT [35]. The GqPCRs function primarily via activation of phospholipase C-β [36], which further produces inositol 1,4,5 trisphosphate and diacylglycerol. These second messengers elevate protein kinase C (PKC) activity, and affect the AKT/PI3K [37] and other signaling pathways [38] to induce the GqPCR effects [39–42]. Two unexpected GqPCR-induced physiological outcomes are cell cycle arrest and apoptosis [43]. For instance, cardiac hypertrophy is mediated by mediators acting through GqPCRs [44]. In addition, gonadotropin-releasing hormone (GnRH) induces apoptosis in granulosa [45] and prostate cancer [46] cells, and prostaglandin F2α (PGF2 α) induces apoptosis of granulosa cells [47], both by reduced AKT activity.

In order to further study the mechanism of GqPCR-induced inactivation of AKT, we previously screened 21 cell lines and found a stimulated reduction in AKT phosphorylation in 10 of them [48]. This effect was PKC-dependent, correlated with reduced AKT activity, JNK activation, and in some cases led to apoptosis. We showed that the apoptosis is mediated by two signaling branches, converging at the level of MLK3, upstream of JNK. One branch consists of c-Src activation of MLK3, and the second includes reduction in AKT activity that alleviates its inhibitory effect on MLK3. This study presented a general mechanism that mediates a GqPCR-induced, death receptors-independent, apoptosis [48]. The mechanism of AKT inactivation downstream of GqPCR/PKC, however remained unknown.

Here we show that the main regulator of the stimulated AKT dephosphorylation is PP2A, which switches its interaction from PI3K to AKT upon GqPCR stimulation (PP2A switch). In resting cells, PP2A interacts with the lipid kinase PI3K to dephosphorylate its inhibitory pSer608 of the regulatory p85 subunit (Ser608-p85) [49], thus maintaining high basal PI3K/AKT activity. Upon GqPCR stimulation, PP2A detaches from PI3K and allow its autoinhibition. PP2A then binds to AKT, dephosphorylating its activatory sites and inhibiting its activity. This switch of PP2A from PI3K to AKT upon stimulation is regulated by IGBP1, that directs the PP2Ac to both PI3K and AKT. Although PP2Aa can interact with the IGBP1-PP2Ac complex upon stimulation, it does not seem involved in this switch’s activities. Overall, our results delineate a mechanism of stimulated inactivation of both PI3K and AKT to induce a death-receptors-independent apoptosis in various cells.

## Methods

### The aim, design and setting of the study

The aim of this study was to elucidate the mechanism by which GqPCR induces AKT-inactivation mediated apoptosis. In order to do so, we followed the activity and phosphorylation of AKT, PI3K and identified PP2A as the main regulator of the process. We also found that the PP2A effect is mainly mediated by IGBP1.

### Reagents and antibodies

GnRH analog (GnRH-a), Tetradecanoylphorbol acetate (TPA), Okadaic Acid, Polyethylenimine (PEI), 4′6-diamino-2-phenylindole (DAPI) and PLA kit were obtained from Sigma (Rehovot, Israel). GF109203x was obtained from Calbiochem (Darmstadt, Germany). Protein A/G beads were obtained from Santa Cruz Biotechnology (Santa Cruz, CA, USA). Dharmafect was obtained from Thermo Scientific (Lafayette, CO, USA). 8-iso PGF2 α was purchased from Cayman Chemical (Ann Arbor, MI, USA). Monoclonal anti-PP2A Ab was obtained from BD Transduction Laboratories (New Jersey, USA). Anti-IGBP1 and monoclonal anti-PI3K Abs were obtained from Abcam (Cambridge, UK). Anti-GFP and anti-HA Abs were obtained from Roche Diagnostics (Mannheim, Germany). Monoclonal anti-AKT, Tubulin, GAPDH, and PKC α were obtained from Santa Cruz Biotechnology (CA, USA). Abs to phosphorylated JNK (pJNK), general JNK1/2 (gJNK), general AKT (gAKT) and histone H1 were from Sigma (Rehovot, Israel). Anti-phospho AKT (pS473AKT) was obtained from Cell Signaling Technology (Boston, MA, USA). Anti-PI3K was purchased from Upstate (Lake Placid, NY New York), or Millipore (Billerica, MA). The anti-phosphorylated PI3K (P85-S608) Abs was prepared by the Ab unit of the Weizmann Institute of Science (Rehovot Israel as previously described [50, 51]). Secondary Ab conjugates were from Jackson Immunoresearch (West Grove, PA, USA).

### Buffers

Buffer A: 50 mM β-glycerophosphate (pH 7.3), 1.5 mM EGTA, 1 mM EDTA, 1 mM dithiothreitol, and 0.1 mM sodium vanadate. Buffer H: 50 mM β-glycerophosphate, pH 7.3, 1.5 mM EGTA, 1 mM EDTA, 1 mM DTT, 0.1 mM sodium vanadate, 1 mM benzamidine, 10 µg/ml aprotinin, 10 µg/ml leupeptin, and 2 µg/ml pepstatin A. Coimmunoprecipitation (CoIP) buffer: 20 mM HEPES pH 7.4, 2 mM MgCl_2_, 2 mM EGTA, 150 mM NaCl and 0.1% Triton.

### Plasmids

PP2Ac plasmid was cloned from mRNA from HeLa cells into HA-pCDNA3 between the HindIII and BamHI sites. PP2Aa cDNA in pMIG was obtained from Addgene, and transferred into pEGFPC1 between EcoRI and KpnI with FLAG preserved. The mutants was prepared using Quickchange method. IGBP1 was cloned from HeLa cells and the GFP-IGBP1 plasmid was created in pEGFP plasmid using BamH1 sites in both sides.

### Cell culture and transfection

αT3 cells were obtained and cultured as previously described [52]. Briefly, the cells were cultured in Dulbecco’s modified Eagle’s medium (DMEM) supplemented with 2 mM L-glutamine, 1% pen/strep and 10% fetal bovine serum (FBS). SVOG4 cells from N. Auersperg (University of British Columbia, Vancouver, Canada) were cultured in the same medium combination with the addition of hydrocortisone (0.5 mg/ml), and Gentamycin Hydrochloride. PC3 cells from ATCC were cultured in RPMI supplemented with 2 mM L-glutamine, 1% pen/strep and 10% FBS. Cells were transfected using polyethylenimine (Sigma; Rehovot Israel [53]). Si-RNAs were transfected using Dharmafect (Dharmacon) according to the manufacturer’s instructions.

### Cell extraction and western blotting

Cells were grown to subconfluency and then serum-starved (0.1% FBS for 16 h, as described [54, 55]. After stimulation or other treatments, cells were rinsed twice with ice-cold phosphate buffered saline (PBS), which was replaced with Buffer H. The cells were then scraped into Buffer H (0.5 ml/plate), sonicated (50 W, 2 × 7 s), and centrifuged (20,000 × g, 15 min). Aliquots of cellular extracts were subjected to SDS-PAGE and transferred onto nitrocellulose membranes (Tamar, Jerusalem, Israel) by electroblotting. Membranes were incubated with the corresponding primary Ab (60 min, 23 °C), followed by washes and incubation with horseradish peroxidase conjugated secondary Ab. The phospho and general Abs were probed on distinct blots with equal amount of loading. Blots were developed using the ChemiDoc (BioRad, Hercules, CA USA). Each experiment was performed at least three times to obtain significant data. Quantification of the band intensities was performed using BioRad analysis tool (Madison WI, USA).

### Non-denaturing gel electrophoresis

Cells were grown to subconfluency, starved (0.1% serum) for 16 h, harvested in Buffer H not containing DTT, sonicated and centrifuged (20,000 × g, 15 min, 4 °C). Samples were resuspended in non-denaturing sample buffer (62.5 mM Tris–HCl, pH 6.8, 25% glycerol, 1% Bromophenol Blue) and resolved on 8% native gel (without SDS and DTT in both the polyacrylamide gel and the running buffer). Western blot was performed as described above. Molecular weight markers were resolved in the gel were the same as those used for SDS gels.

### Coimmunoprecipitation

Cells were grown to subconfluency, serum-starved as above, and treated as indicated. Cell extracts were produced as previously described [55] and incubated for 2 h at 4 °C with Protein A/G-agarose beads (Santa Cruz Biotechnology, CA, USA) pre-linked with specific Abs (1 h, 23 °C). The bound A/G beads were washed three times with ice-cold washing buffer containing 10 mM Tris, pH 7.4, 1 mM EDTA, 1 mM EGTA, pH 8.0, 150 mM NaCl, and 0.5% Triton X-100. Beads were then resuspended with 1.5X sample buffer and boiled; the resolved proteins were analyzed by Western blotting with the indicated Abs.

### PI3K activity

PI3K activity was determined as previously described [56]. Briefly, serum-starved cells were stimulated, washed and then lysed in PI3K lysis buffer (20 min on ice). The lysates were then, centrifuged (15,000xg, 15 min, 4 °C), and incubated with immobilized (Protein A/G-agarose beads) anti-p85α Ab (1 h, 37 °C). Co-IP (2 h at 4 °C), followed by two washes with lysis buffer and one with Tris-buffered saline: 50 mM Tris–HCl, pH 7.4, 150 mM NaCl. PI3K activity was assayed by resuspending the IP in the same buffer (50 μl) with sonicated phosphoinositides (L-α-phosphatidyl-D-myo-inositol; final concentration, 0.2 mg/ml) and [γ-^32^P]ATP (Amersham Biosciences, UK; 40 μM; 12,000 cpm/pmol). The kinase reactions (10 min, 37 °C) were stopped by 200 μl of 1 N HCl and 400 μl of CHCl3/MeOH (1:1). The organic phase was collected and re-extracted with 40 μl of MeOH/HCl (1:1). The samples were then dried, resuspended in 30 μl of CHCl3/MeOH (1:1), and spotted onto Silica Gel 60 TLC (GE, Pittsburgh, PA USA). The plates were developed in propanol/2 M acetic acid (65:35), and autoradiographed. Phospholipid markers (Sigma Chemicals Co., St. Louis, MO) were used for the identification of the products.

### TUNEL

Apoptosis analysis was done as previously described [57]. Subconfluent PC3 and αT3-1 cells were plated on glass coverslips in 12 well plates under the standard culture conditions as described above. Twenty-four hr after the initial seeding, cells were serum-starved and then treated. At different times after treatment the cells were fixed with paraformaldehyde solution (4% in PBS (pH 7.4) for 1 h at 23 °C), washed with PBS and then incubated with 0.1% Triton X-100 in 0.1% sodium citrate (2 min, 4 °C), washed again with PBS, and incubated with terminal deoxynucleotidyltransferase-mediated nick end labeling (TUNEL) reaction mixture containing fluorescein-dUTP and terminal deoxynucleotidyltransferase (Roche Molecular Biochemicals, Germany) for 30 min at 37 °C. Preparations were analyzed by fluorescence microscopy.

### Proximity ligation assay

Protein–protein interactions were detected with Duolink PLA Kit (Olink Bioscience), according to the manufacturer’s protocol as described [58]. Briefly, cells were grown, fixed and permeabilized as described for immunofluorescence staining. The samples were then incubated with primary Abs against two proteins suspected to interact (60 min, 23 °C), and then incubated with specific probes according to manufacturer’s protocol, followed by DAPI staining to allow visualization of nuclei. The signal was visualized as distinct fluorescent spots by fluorescence microscopy (Olympus BX51, or spinning disc confocal Zeiss microscope both at × 40 magnification). Background correction, contrast adjustment and quantification were performed using Photoshop (Adobe) and ImageJ.

### Statistical analysis

Data are expressed as mean ± S.E, carried out using Student’s t test (two-tailed) to test for differences between the control and experimental results.

## Results

### PP2A regulates the PKC-dependent AKT inactivation

**Fig. 1:**
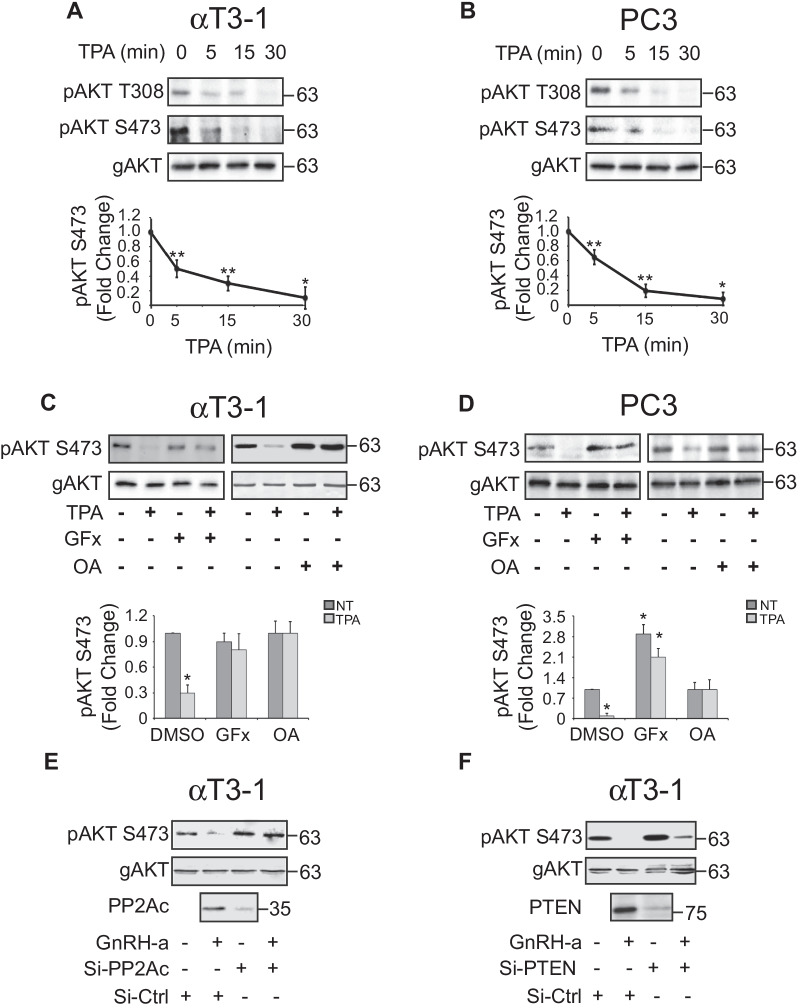
PKC activation induces PP2A-dependent AKT dephosphorylation. **a**, **b** TPA induces AKT dephosphorylation**.** Serum-starved (16 h, 0.1% FCS) αT3-1 (**a**) or PC3 (**b**) were stimulated with TPA (250 nM) for the indicated times. Then the cells were harvested, and cell extracts were subjected to Western blotting with Abs to p473AKT (pAKT) and general AKT (gAKT). **c, d** The TPA-induced AKT dephosphorylation is mediated by PKC and PP2A. Serum-starved αT3-1 (**c**) or PC3 (**d**) cells were pretreated with either the PKC inhibitor GFx (GF, 3 µM, 20 min) or the PP2A inhibitor okadaic acid (OA, 0.5 µM20 min) prior to TPA stimulation (250 nM, 30 min) and blots were analyzed as described above. Quantification of three experiments is presented in the bottom graphs (*p < 0.01, **p < 0.05). **e** Knockdown of PP2Ac inhibits the PKC-dependent AKT dephosphorylation. αT3-1 cells were transfected with Si-RNAs of PP2Ac (both PP2Ac isoforms), serum-starved, stimulated with GnRH-a (0.1 µM, 30 min) and then harvested. pAKT and PP2Ac expression were determined by the indicated Abs. Knockout efficiency (bottom panel) was determined by reblotting the same membrane with PP2Ac Abs after stripping. **f** Knockdown of PTEN does not affect the PKC-dependent AKT dephosphorylation. αT3-1 cells were transfected with Si-RNA of PTEN, serum-starved, stimulated with GnRH-a (0.1 µM, 30 min) and then harvested. pAKT and PP2Ac expression were determined by the indicated Abs. Knockout efficiency was determined using Western blotting with the indicated Abs. Knockout efficiency (bottom panel) was determined by reblotting the same membrane with PTEN Abs after stripping

We have previously shown that in several cell types, activation of PKC, either by GqPCR or directly by phorbol ester (TPA) results in an inhibition of AKT phosphorylation on its activatory residues Thr308 and Ser473 [48]. PGF2α and GnRH-a were shown to decrease basal AKT phosphorylation and activity. We undertook to study the mechanism of this unique signaling event, and for this purpose chose three cell lines that demonstrated stimulation-dependent decrease in AKT phosphorylation: the pituitary αT3-1, granulosa SVOG-40 and prostate cancer PC3. Most of the experiments below were performed on two out of these three cell lines. As expected [48], PKC activation by either TPA (Fig. 1a, b) or GqPCR ligand (PGF2α; Additional file 1: Fig. S1A,B) reduced the phosphorylation of the activatory pThr308 and pSer473 residues of AKT in a time dependent manner. The dephosphorylation of both phosphorylated residues was very similar to each-other, which is in agreement with our previous finding in which we found that Ser473 and Thr308 are phosphorylated/dephosphorylated simultaneously under all conditions used [48]. Throughout the rest of the study we show mainly the effect on pSer473, which generally represents pThr308 as well. As expected, inhibition of PKC activation using the pan-inhibitor GF109203x (GFx) prevented the reduction in phosphorylation (Fig. 1c, d, and Additional file 1: Fig. S1A). This dephosphorylation of AKT may be catalyzed by PTEN, PP2A, or PHLPP [6–8, 59]. Preincubation with the PP2A inhibitor okadaic acid prevented the dephosphorylation of AKT (Fig. 1c, d and Additional file 1: Fig. S1B), and the effect of PP2A was confirmed by knockdown of the PP2Ac in αT3-1 cells (Fig. 1e). No effects were observed with SiRNA of the lipid phosphatase PTEN (Fig. 1f). These results, together with the fact that PC3 are PTEN-null cells [60], indicates that PTEN or PHLPP, which is not sensitive to okadaic acid, are not major regulators of this process.

### PP2A-regulated PI3K inactivation plays a role in AKT dephosphorylation

**Fig. 2:**
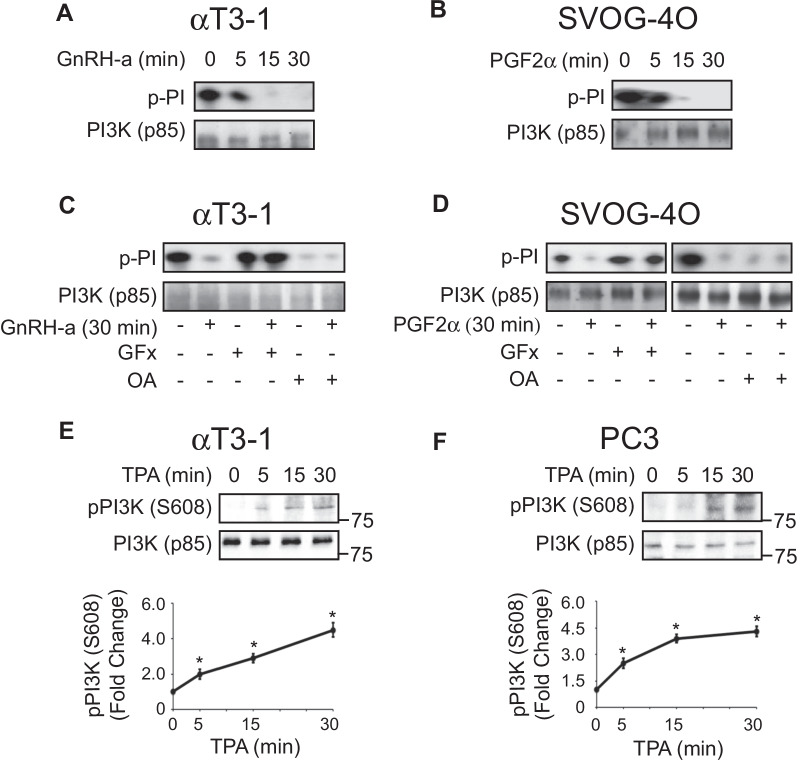
PKC and PP2A-dependent PI3K inactivation by autophosphorylation of Ser608-p85. **a, b** Gq-dependent inactivation of PI3K. Serum starved αT3-1 (**a**) and SVOG-40 (**b**) cells were stimulated with 0.1 µM GnRH-a or 10 μM PGF2α for 30 min. The cells were then harvested, and PI3K was IPed with anti-PI3K (p85) Ab, followed by PI3K lipid phosphorylation assay. The amounts of PI(3)P (p-PI) produced were detected by autography (upper panels), and the amount of p85 IPed was detected by anti-p85 Ab (lower panels). **c, d** The inactivation of PI3K is dependent on PKC and PP2A. Serum starved αT3-1 (**c**) and SVOG-40 (**d**) cells were pretreated with either the PKC inhibitor GFx (GF, 3 µM, 20 min) or the PP2A inhibitor okadaic acid (OA, 0.5 µM, 20 min), treated with 0.1 µM GnRH-a or 10 μM PGF2α for 30 min, and then subjected to lipid phosphorylation assay as in **a**, **b**. **e, f** Anti-phospho Ab reveals a PKC dependent phosphorylation of Ser608-p85. Serum starved αT3-1 (**e**) or PC3 (**f**) cells were stimulated with TPA (250 nM) for the indicated time, cells were harvested and analyzed by Western Blotting with the newly developed anti-pPI3K (pSer608-p85) and anti-p85 Abs. The graphs in the bottom panels represent means ± standard errors of three experiments. * p < 0.01

In view of the dephosphorylation of AKT upon stimulation, we undertook to elucidate the upstream components that may contribute to the effect. Since PI3K is the main activator of AKT [61], we first examined its involvement. To our surprise, we found that in resting cells, PI3K has a pronounced basal activity, which is decreased shortly after stimulation with PGF2α or GnRH-a and abolished within 30 min (Fig. 2a, b). Due to their involvement in AKT regulation, we then examined whether PKC and PP2A are involved in the downregulation of PI3K activity as well. Indeed, we found that GFx prevented the stimulus-induced inactivation (Fig. 2c, d), suggesting that unlike most systems, PKC is involved in the PI3K regulation. More surprising, however, was the finding that treatment of quiescent SVOG-40 and αT3-1 cells with okadaic acid results in a significant reduction of the basal catalytic activity of PI3K (Fig. 2c, d). This effect was not changed upon stimulation, suggesting that PP2A plays a role in the maintenance of the relatively high basal PI3K activity, without much effect on the stimulated activity. Interestingly, it has previously been shown that the catalytic 110 kDa subunit of PI3K is not just a lipid kinase, but has an intrinsic protein Ser/Thr kinase activity as well [49, 62]. Thus, the 110 kDa subunit is capable of autophosphorylating Ser608 of the PI3K’s p85α subunit, which results in a decreased PI3K activity, and PP2A reverses this inhibitory effect [62, 63]. To test whether such an effect is involved in the regulation of PI3K in our system, we generated antibody (Ab) to pSer608-p85, and confirmed its specificity to the phosphorylated residue (Additional file 1: Fig. S2). Indeed, the Ab detected elevated p85 phosphorylation upon PKC activation by TPA or GnRH and okadaic acid (Fig. 2e, f and Additional file 1: Fig. S2). Thus, our results confirm the phosphorylation of Ser608 on p85, indicate that it operates in our system, and suggest that it might be the site of the PP2A-mediated regulation.

### PP2A detaches from PI3K and binds to AKT upon stimulation to form a PP2A switch

**Fig. 3:**
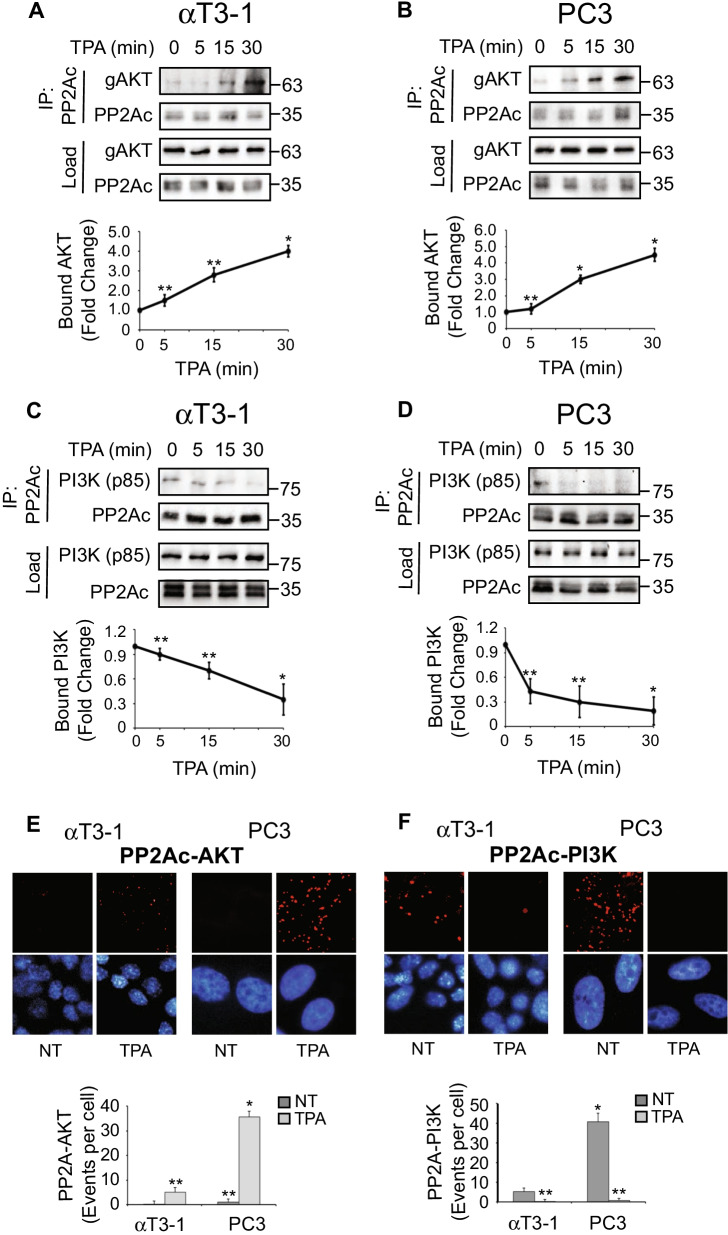
PP2Ac is detached from PI3K and interacts with AKT upon stimulation. **a, b** Increased AKT-PP2Ac interaction upon stimulation. Serum starved αT3-1 (**a**) and PC3 (**b**) cells were stimulated with TPA (250 nM) for the indicated times and harvested. Then, PP2A was IPed using anti-PP2A and the CoIPed AKT was detected using the indicated Abs. The graphs in the bottom panels represent means ± standard errors of three experiments. * p < 0.01, ** p < 0.05. **c, d** PP2Ac interacts with PI3K’s p85 in resting cells and the interaction is decreased upon stimulation. Serum starved αT3-1 (**c**) and PC3 (**d**) cells were stimulated with TPA (250 nM) for the indicated times and harvested. Then, PP2A was IPed using anti-PP2A and the CoIPed p85 was detected using the indicated Abs. The graphs in the bottom panels represent means ± standard errors of three experiments. * p < 0.01, ** p < 0.05. **e, f** Using PLA to follow PP2Ac interaction with AKT and PI3K’s p85. αT3-1 (left) and PC3 (right) cells were cultured on cover slips. The cells were then serum starved, stimulated with TPA (250 nM, 30 min) and fixed. Protein–protein interactions were detected and quantified using the PLA kit, as described in experimental procedures. The bar graphs in the lower panels represent means ± standard errors of a representative experiment that was reproduced 3 times. Significance of change from non-stimulated (NT) is calculated. * p < 0.01, ** p < 0.05

Since dephosphorylation often requires direct interactions between the phosphatase and its target [64], and to further learn about the role of PP2A in the regulation of PI3K and AKT, we undertook to study the interactions of the phosphatase with the two kinases. For this purpose, we performed coimmunoprecipitation (CoIP) experiments and found that the catalytic subunit of PP2A (PP2Ac) specifically precipitates with AKT. This interaction was significantly enhanced upon TPA stimulation in both cell lines (Fig. 3a, b). We then examined whether PP2A may interact with PI3K, and whether this interaction can be modulated by stimulation. Thus, both cell lines were subjected to CoIP, using anti-PP2Ac Ab for the immunoprecipitation (IP) and anti-PI3K Ab for Western blotting. This experiment revealed that PI3K interacts with PP2A in quiescent cells, and this interaction is significantly reduced after stimulation (Fig. 3c, d; Additional file 1: Fig. S3). The results of both PP2Ac-AKT and PP2Ac-PI3K interactions were confirmed using additional cell lines and stimuli as well as proximity ligation assay (PLA; Fig. 3e, f; Additional file 1: Fig. S4). Our results best fit a model in which in resting cells, PP2A interacts with PI3K, dephosphorylates the inhibitory pSer608-p85, and thus keeps the PI3K active. Upon stimulation, PP2A is detached from PI3K, and thereby allows incorporation of phosphate to Ser608-p85 by autophosphorylation, inactivating the kinase. The PP2A released from PI3K then interacts with AKT, and dephosphorylates the activatory Thr308 and Ser473 residues of the kinase. We termed these alternate interactions that downregulate both PI3K and AKT activities “PP2A switch”.

### IGBP1 regulates the PP2A switch

**Fig. 4:**
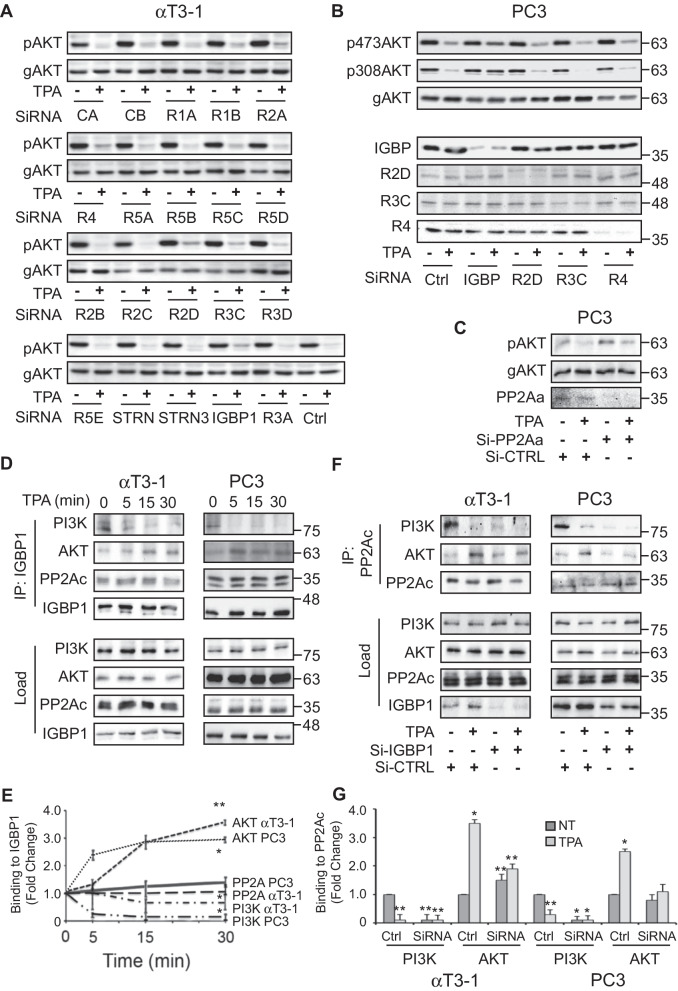
Identification of IGBP1 as a key regulator of the PP2A switch. **a** Si-RNA screen to identify the PP2A B subunit involved in the PP2A switch. αT3-1 cells were transfected with the indicated Si-RNAs by incubation of two 6 cm plates for each Si-RNA (6 h, 20 nM SiRNA pool). Then, the cells were transferred to a fresh medium with 10% FCS, and the cells were left for 24 h recovery followed by serum starvation for 14 h. Next, for each SiRNA, the cells in one plate were stimulated with TPA (250 nM, 15 min) and the other plate was left untreated. Finally, the cells were lysed and the extracts were subjected to Western blotting with the indicated Abs. The MW of the gAKT and pAKT in all lanes is 64 kDa. **b** SiRNA of IGBP1 reduces the stimulated AKT dephosphorylation in PC3 cells. PC3 cells were transfected with the indicated SiRNA and treated as described for the αT3-1 cells in A. In the final stage, the cells were subjected to Western blotting with additional Abs as indicated. **c** Combined SiRNAs of the two PP2Aa subunits does not affect AKT dephosphorylation. PC3 cells were transfected with the combined SiRNA of both PP2Aa isoforms or scramble control (Si-CTRL) for 6 h. Forty-eight hr later, the cells were serum-starved (16 h) and then stimulated with TPA (250 nM, 30 min, +) or left untreated (-). Cell extracts of each treatment were then subjected to Western blotting using the indicated Abs. **d** Stimulated IGBP1 interactions with PI3K’s p85 (PI3K), AKT and PP2Ac. αT3-1 (left panels) or PC3 (right panels) cells were serum starved and stimulated with TPA (250 nM) for the indicated times. The cells were harvested and then IGBP1 was IPed from the cell extracts using anti-IGBP1 Ab. CoIPed proteins as well as the proteins in the loaded extracts were detected using Western blotting with the indicated Abs. **e** Quantification of the results in **d**. The graphs represent the average and standard errors of three independent experiments. Significance of change relative to non-stimulated (NT) control is calculated for each protein and cell line. * p < 0.01, ** p < 0.05. **f** IGBP1 is required for the interaction of PP2A with PI3K and AKT. αT3-1 (left panels) or PC3 (right panels) were transfected with the SiRNA (75 nM) of IGBP1 or Scramble control (CTRL) for 6 h (50 nM SiRNA pool), after which the cells were moved to medium containing 10% FCS for 48 h to recover followed by serum starvation for additional 16 h. Then the cells were stimulated with TPA (250 nM, 30 min), harvested, and PP2A was IPed from the cell extracts using anti-PP2A. The CoIPed proteins were detected using Western blotting with the indicated Abs. **g** Quantification of the results in **E**. The results shown represent means ± standard errors of three experiments. Significance of change from non-stimulated (NT) control is calculated for each protein and cell line. * p < 0.01, ** p < 0.05

PP2A usually acts as a heterotrimeric complex composed of PP2Aa, PP2Ac and a regulatory B subunit. While the A and C subunits are each encoded by two distinct genes that are very similar to each other, there are no less than 17 different B subunits and several other PP2Ac-interacting proteins that contribute to the specificity of the phosphatase [12]. To determine the specific subunit(s) that regulates the PKC-dependent AKT dephosphorylation, we performed an SiRNA screen in αT3-1 cells. In this screen, we knocked down the expression of most types of B, as well as IGBP1, the two PP2Ac and the two PP2Aa isoforms, followed by stimulation with GnRH-a for 30 min (Fig. 4a). A loss of stimulation-dependent pSer473-AKT dephosphorylation that was consistent in the screens performed was observed with the SiRNA of R2D, R3C and, most prominently, IGBP-1. Unlike the results with the knockdown of the two isoforms of PP2Ac together (Fig. 1e), the individual knockdown of each PP2Ac isoform (Fig. 4a) did not affect the stimulation-dependent AKT dephosphorylation. The reason is probably that the reduction in total expression of PP2Ac did not exceed 50%, which is not sufficient to cause cellular effects. Although it is possible that the knockdown of some proteins was not sufficient, and they do participate in AKT regulation, we decided to concentrate on the B-subunits that did show some effect. Therefore, we examined the effect of these SiRNAs (Fig. 4b), as well as the SiRNA of R4 (PTPA), on the stimulated AKT dephosphorylation in PC3 cells. Although all four SiRNAs reduced the expression of their cognate proteins (IGBP1 – 85%, R2D – 90%, R3C—60%, and R4—95%), reduction of the stimulated pSer473-AKT dephosphorylation was observed only with the SiRNA of IGBP1 (Fig. 4b). This effect was even more pronounced with pThr308-AKT, indicating that IGBP1 is the main subunit responsible for the PP2A effect on both phosphorylated residues in our system. The lack of effect of the knockdown of each individual C subunit (Fig. 4a) could be due to insufficient reduction of the expression of this protein. We therefore used combined knockdown of the two isoforms of PP2Aa in PC3 cells. We found that significant reduction (~ 75%) in the total expression of PP2Aa had no significant effect on AKT dephosphorylation (Fig. 4c). These results indicate that unlike PP2Ac and IGBP1, PP2Aa is not involved in the PP2A switch.

### IGBP1 directs PP2A to PI3K in resting cells and to AKT upon stimulation

**Fig. 5:**
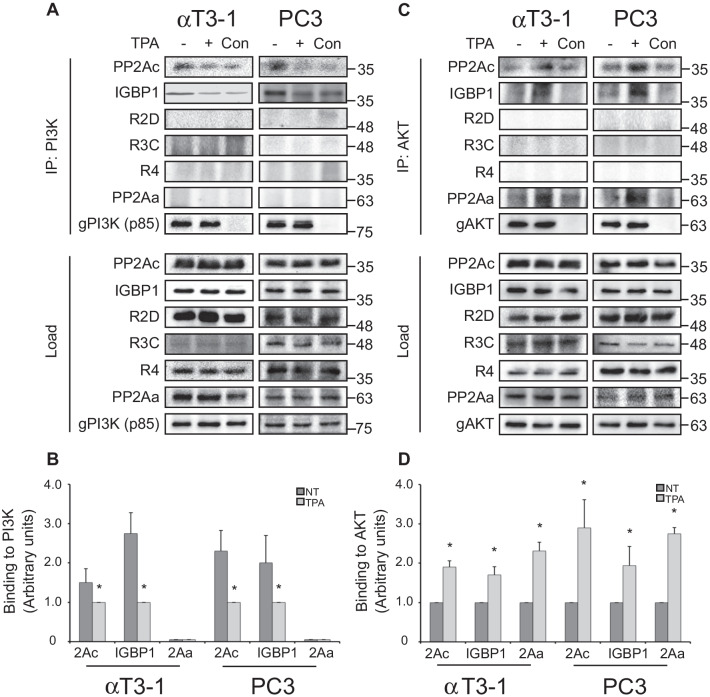
CoIP of PP2A components with PI3K and AKT. **a** CoIP of the PP2A components with PI3K. Serum starved αT3-1 and PC3 cells were either stimulated with TPA (250 nM, 30 min; + , Con) or left untreated as control (-) and then harvested. Then, anti PI3K Ab or non-relevant Ab (anti histone H1, Con) were used for IP, and the CoIPed proteins, as indicated, were detected by the relevant Abs. **b** Quantification of the results in A. The bar-graph represent means ± standard errors of three experiments for the indicated proteins.* p < 0.01, ** p < 0.05. **c** CoIP of the PP2A components with AKT. Serum starved αT3-1 and PC3 cells were either stimulated with TPA (250 nM, 30 min; + , Con) or left untreated, and then harvested. Then, anti AKT Ab or non-relevant Ab (anti KRas, Con) were used for IP, and the CoIPed proteins were detected by the indicated Abs. **d** Quantification of the results in C. The bar-graphs represent means ± standard errors of three experiments of the indicated proteins.* p < 0.01, ** p < 0.05

In order to follow the mechanism by which IGBP1 regulates the PP2A switch, we first examined its interaction with PI3K, AKT and PP2Ac. CoIP experiments using IGBP1 Ab in both αT3-1 and PC3 revealed that in resting cells, this protein is bound to PI3K (p85 subunit) and PP2Ac, but not to AKT. After TPA stimulation, the interaction with PI3K diminished, the interaction with PP2Ac did not change, and the interaction with AKT increased (Fig. 4d, e). These results were confirmed using PLA (Additional file 1: Fig. S4), indicating that IGBP1 is primarily responsible for recruiting the switch-related PP2A complex. To further verify this point, we used Si-RNA of IGBP1 and followed the effect of the knockdown on the interaction in both αT3-1 and PC3 cells. Our results show that specific knockdown of IGBP1 (see reduced IGBP expression and rescue by overexpression of GFP-IGBP1 (Additional file 1: Fig. S5)) abolished PP2Ac-PI3K interactions, independent of stimulation, and also altered the stimulated PP2Ac binding to AKT (Fig. 4f, g). To further confirm these results and learn more about the components involved, we employed the reciprocal CoIP experiment, using either PI3K or AKT for the IP step. As expected from the experiments above, we found that the binding of PP2Ac and IGBP1 to PI3K is decreased upon TPA stimulation (Fig. 5a, b), while their binding to AKT is increased (Fig. 5c, d). On the other hand, the B subunits that might have been involved in the reaction according to the αT3-1 screen (R2D, R3A, R4; Fig. 4a) did not bind to AKT or PI3K under any of the conditions used. This lack of binding of other B subunits to AKT was confirmed also by mass spectrometry (Additional file 1: Table S1). These results imply that IGBP1 has a dual function in the PP2A switch. One is the anchoring of a PP2Ac to PI3K in resting cells, and the other is the stimulated binding of the PP2Ac to AKT. These functions fully inactivate AKT, and thereby mediate its downstream effects.

### Stimulated interaction of PP2Aa with the IGBP1-PP2Ac complex is not involved in the PP2A switch

**Fig. 6:**
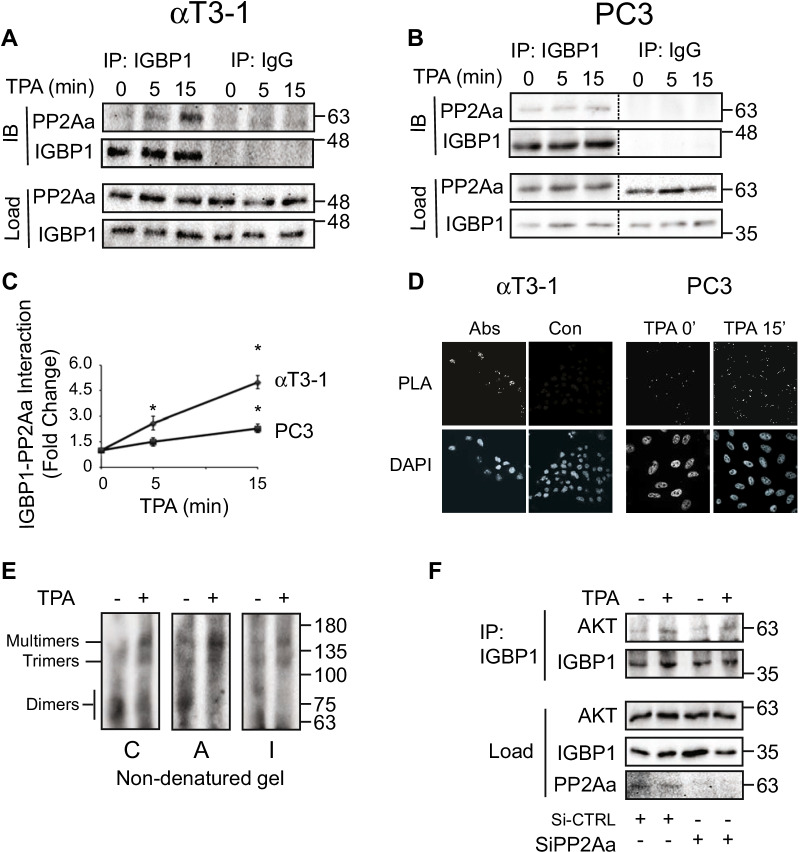
Interaction of IGBP1 with PP2Aa. **a, b** IGBP1 interacts with PP2Aa with and without stimulation. Serum-starved αT3-1 **a** or PC3 **b** cells were treated with TPA (250 nM) for the indicated times. Then, anti IGBP1 Ab or non-relevant Ab (non-relevant IgG, Con) were used for IP, and the CoIPed proteins were detected with PP2Aa Ab. The amount of IPed IGBP as well as PP2A and IGBP loading were detected using with the indicated Abs. **c** Quantification of the results in **a**, **b**. The graphs represent means ± standard errors of three experiments for the indicated proteins.* p < 0.01. **d** PLA verifies PP2Aa interaction with IGBP1. αT3-1 (left) and PC3 (right) cells were cultured on cover slips. The cells were then serum starved, and either stimulated with TPA (250 nM, 15 min) or left untreated (both panels of αT3-1 cells and TPA 0 in PC3 cells) followed by fixation. Protein–protein interactions of PP2Aa and IGBP1 were detected using the PLA kit with their cognate Abs as described under Experimental Procedures. The control in the αT3-1 cells means that no PP2Aa Ab was used in the reaction. **e** Non-denaturing gel electrophoresis to detect PP2A complexes. Cells were grown to 70% confluence, starved for 16 h, and then were either stimulated with TPA (250 nM, 30 min; +) or left untreated (-). The formation of PP2A complexes and whether they contain PP2Ac (**c**), PP2Aa (**A**), and IGBP1 (I) was detected by non-denaturing gels, followed by blotting with the indicated Abs. The lower molecular weight proteins are not presented here. The molecular weight standard is shown here although the migration of the marker proteins in this native gel does not reflect the actual mass as it depends on the charge of each protein as well. Thus the position of the markers provides just a rough estimation of the actual molecular weight. **f** PP2Aa does not affect AKT interaction with IGBP1. The same extracts from Fig. 4C were taken for additional experiments and subjected to IP with anti IGBP1 Ab. The CoIPed and IPed proteins and loading extracts were detected using Western blotting with the indicated Abs

It was previously reported that IGBP1 may interact not only with PP2Ac, but also with PP2Aa, forming different sets of dimers [19]. In order to examine this point, we blotted the CoIPed proteins with anti PP2Aa Ab. While the PP2Aa subunit did not interact with PI3K at all (Fig. 5a, b), it did interact with AKT upon TPA stimulation (Fig. 5c, d). This result was confirmed by mass spectrometry analysis that showed interaction of PP2Aa with both AKT and IGBP1 (Additional file 1: Table S1). The fact that all three PP2A subunits examined, namely PP2Ac, PP2Aa and IGBP1, bind to AKT upon stimulation (Fig. 5) may indicate that the three subunits form complexes. However, since the ability of PP2Ac to interact simultaneously with IGBP1 and PP2Aa is controversial ([15, 19] vs [20, 21]), we undertook to examine it in our system. For this purpose, we first CoIPed IGBP1 with PP2Aa before and after stimulation, in both αT3-1 and PC3 cell lines. Our results demonstrate that in resting cells, the interaction between the two subunits is negligible, but is significantly elevated upon TPA treatment (Fig. 6a–c). This was confirmed by PLA, as we detected a specific interaction in resting cells (Fig. 6d) that was increased after stimulation (Fig. 6d right panel). Finally, mass spectrometry verified the presence of the PP2Aa as well as PP2Ac in the IGBP1 CoIP of PC3 cells (Additional file 1: Table S1).

Although our results clearly indicate that IGBP1 can bind to both PP2Aa and PP2Ac after stimulation, they do not verify formation of a heterotrimer between these subunits. We therefore resorted to non-denaturing gel electrophoresis in order to examine whether a trimeric PP2A complex may be detected after stimulation. In resting cells, all three subunits were found mainly in various dimers ranging in their molecular weights between ~ 55 to ~ 80 kDa, although higher molecular weight complexes were seen as well (Fig. 6e). After TPA stimulation, the dimers disappeared and shifted to increased levels of trimers (~ 130 kDa) and mainly other multimers (160–180 kDa). The molecular weight of the trimers may indicate the existence of a PP2Aa, PP2Ac and IGBP1-containing holoenzyme, while the multimers likely contained the holoenzyme bound to its substrate(s). Complexes containing PI3K could not be detected in our system because of their very high mass. Importantly, when PP2Aa was knocked-down, it did not affect the IGBP1-AKT interaction (Fig. 6f), indicating that the interaction of these components is not dependent on the scaffolding function of the PP2Aa, and corroborating the lack of effect of PP2Aa knockdown on the PP2A switch shown above (Fig. 4c). Taken together, our results show for the first time that the PP2A composition changes shortly after stimulation. In addition, we show the possible formation of an IGBP1, PP2Aa and PP2Ac-containing holoenzyme. This interaction is stimulus-dependent, indicating that it requires post-translational modifications, which may explain why it is not seen in some systems [19, 21]. However, due to lack of effect of PP2Aa on the binding of PP2Ac to or dephosphorylation of AKT, it seems that PP2Aa is not involved in the PP2A switch, and the IGBP1-PP2Ac dimer is sufficient for its function.

### IGBP1 regulates the PP2A switch-dependent TPA-induced apoptosis

**Fig. 7:**
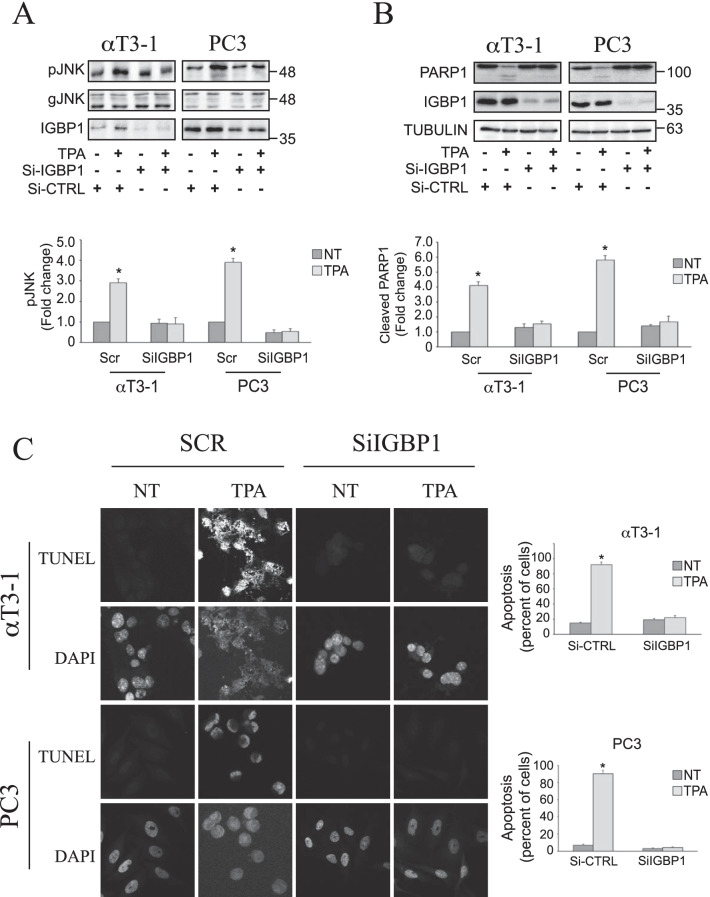
IGBP1 is essential for PP2A-dependent JNK activation and apoptosis upon TPA stimulation. **a** PC3 and αT3-1 cells were treated with scramble (SCR) or IGBP1 SiRNA for 48 h. Cells were then grown to 70% confluency, serum starved (0.1% serum for 16 h) and then treated with or without TPA (250 nM, 30 min). Cells were harvested in RIPA buffer and the cell extracts were separated and immunoblotted with the indicated antibodies. The bar-graphs represent average and standard errors of three distinct experiments. **b** Cells were treated as in A, except that the TPA (250 nM) was added for 48 h. Then the cells were harvested in RIPA buffer and the cell extracts were separated and immunoblotted with the indicated antibodies. The bar-graphs represent average and standard errors of two distinct experiments. **c** Cells were treated as in B followed by fixation with 4% PFA and apoptotic TUNEL assay. The bar-graphs represent average and standard errors of three distinct experiments NT-non-treated. In all cases: * p < 0.01

We show here that the PP2A switch can induce the inactivation of AKT, and this is regulated by IGBP1, which is important both for the release of PP2Ac from PI3K and for its binding to AKT. In order to study whether this IGBP1-dependent PP2A action indeed affects the physiological roles of TPA in the switch-containing cells we undertook to examine whether it may be involved in the TPA-induced JNK activation and apoptosis as reported in our previous study [48]. Thus, we knocked down IGBP1 in both αT3-1 and PC3 (> 80% reduction in all cases), and then treated the cells with TPA for 30 min. As expected, we found that TPA induced the JNK phosphorylation, and this activation was strongly decreased in the IGBP1 reduced cells (Fig. 7a). In order to test the effect on TPA-induced apoptosis in these cells, we used the IGBP1 knockdown cells in two apoptotic assays, namely PARP1 cleavage (Fig. 7b) and TUNEL (Fig. 7b) in which the TPA was administrated for 2 days. Indeed, the IGBP1 knockdown completely prevented the TPA-induced apoptosis as detected by both methods in both cell types. Thus, these results confirm that the TPA-induced JNK activation that leads to apoptosis is mediated by the IGBP1-mediated PP2A switch in both cell lines tested where the switch is active.

## Discussion

**Fig. 8:**
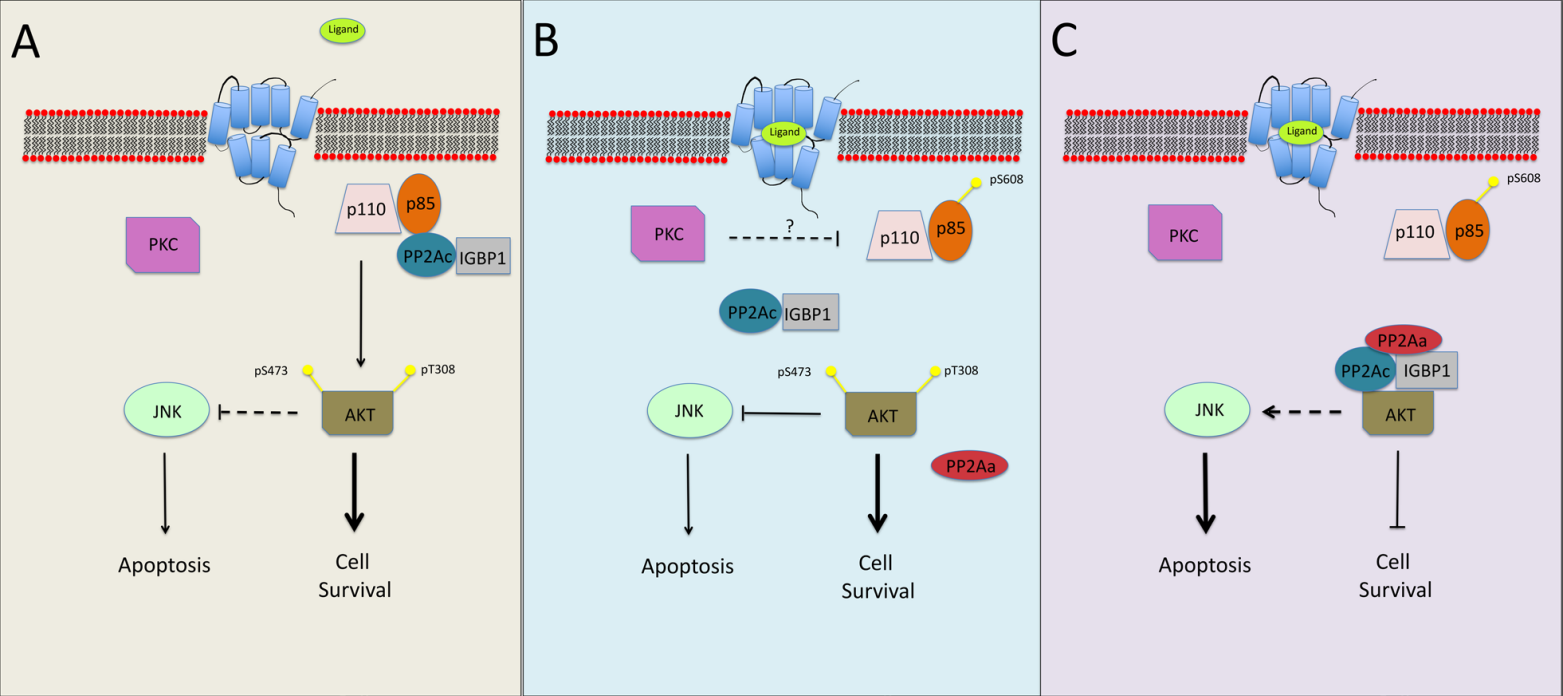
Schematic representation of the PP2A switch. In resting cells (**a**) the PP2Ac-IGBP1 dimer interacts with the p85 subunit of the PI3K, and thereby dephosphorylates it and keeps it active, resulting in the phosphorylation of AKT on both Thr308 and Ser473. The active AKT then phosphorylates MLK3 and thereby blocks the activation of JNK [48]. **b** Upon PKC activation by GqPCR or TPA, the PP2Ac-IGBP1 dimer is detached from PI3K, thus allowing inactivation through autophosphorylation on Ser608. **c** The released dimer forms a trimer with PP2Aa and interacts with AKT, dephosphorylating both the activatory residues of the kinase, thus rendering the AKT inactive, and allow activation of the JNK cascade. A more detailed description is provided in the discussion section

Stimulation of GqPCRs by ligand binding usually results in activation of various intracellular signaling pathways that consequently culminate in the induction of cellular responses. We have previously reported that in some cells, GqPCR stimulation results, instead, in the inactivation of the PI3K-AKT pathway, leading to the induction of the JNK cascade and consequently to apoptosis [48]. This unique signaling seems to play an important role in several physiological and pathological systems, such as cardiac hypertrophy [44], pituitary development [45] and others. However, very little is known about the molecular mechansim involved in the inactivation of the PI3K-AKT pathway within minutes after stimulation, and this is the subject of the current study. Here we undertook to study the molecular mechanisms involved in the inactivation of the PI3K-AKT pathway. Our results best fit a model in which in resting cells, PP2A dimer containing IGBP1 and PP2Ac interacts with PI3K, and dephosphorylates its autoinhibitory pSer608-p85 (Fig. 8). This dephosphorylation results in elevated basal activity of PI3K, formation of PIP3, and activation of AKT. Upon GqPCR stimulation, the PP2A detaches from PI3K and interacts with AKT to form a complex, composed of AKT, PP2Ac, IGBP1, and PP2Aa. The detachment from PI3K results in accumulation of phosphate on Ser608-p85 by autophosphorylation, and therefore, inactivation of PI3K. In parallel, the stimulation leads to an attachment of the PP2A holoenzyme containing PP2Ac, PP2Aa and IGBP1 to AKT, resulting in the dephosphorylation of the regulatory Thr308 and Ser473, and inactivation. We name this process a “PP2A switch”, and show that it is responsible for JNK activation and shift to apoptosis.

We show that one of the main regulators of the PP2A switch is IGBP1. This protein was initially identified as a regulator of Ig receptor signal transduction in B and T lymphocytes [65], but soon after was shown to interact with and regulate PP2Ac, as well as PP4 and PP6 [17, 66]. Although it was shown that IGBP1 might participate in the regulation of specificity of PP2A, it clearly does not behave as a canonical B subunit under most conditions. For example, IGBP1 can form a stable complex with PP2Ac, without the involvement of any PP2Aa or any other B subunits. In fact, it was initially proposed that the binding of IGBP1 and PP2Aa to PP2Ac is mutually exclusive, mainly because the binding of the subunits requires charged residues in adjacent and even overlapping surfaces in PP2Ac [15, 19]. However, other reports indicated that such an interaction may exist, at least under some conditions [20, 21]). In our systems, the AKT-bound (but not PI3K bound) PP2A contain PP2Ac, PP2Aa and IGBP1. This parallel binding of PP2Aa and IGBP1 seems to require post-translational modification, which might be PKC-dependent phosphorylation. Thus, our results show for the first time that under some circumstances, stimulation may change the composition and substrate binding of the PP2A, and consequently its role in regulation of various cellular processes.

Early studies implicated IGBP1 as either a regulator of, or subject to regulation by mTOR signaling [17], which together with other studies, including ours, suggest that IGBP1 is a key regulator of the broad signaling axis of PI3K-AKT-mTOR [1]. However, IGBP1 does not regulate only this pathway, as it was shown as a key regulator of the stress related proteins MEK3 [18], and the ubiquitin E3 ligase midline-1 (Mid1 [67]). Interestingly, the specificity of PP2A towards these two proteins is determined by the IGBP1 without binding of PP2Aa or any B subunits to the IGBP1-PP2Ac dimer. Moreover, this PP2A dimer was shown to act as a regulator of the phosphatase by additional means, including directing Mid1 to induce its degradation [68]. It was also proposed that IGBP1 could act as a chaperon in the formation of a trimeric PP2A holoenzyme [69]. Thus, IGBP1 was suggested to have two distinct regulatory functions: (i) substrate targeting resembling the B-subunit functions, but without PP2Aa and (ii) modulating PP2A folding, composition and stability. In our system we did not detect any change in the PP2A stability, and IGBP1 clearly directed PP2A holoenzyme to either PI3K or AKT. Therefore, we show that IGBP1 can act as a B-like subunit of PP2A holoenzyme, and our results support the notion that it is a key regulator of PI3K-AKT-mTOR signaling.

As mentioned above, PP2A is a known regulator of the PI3K-AKT pathway, dephosphorylating many of its components [1]. Although less studied, it has been shown that PP2A regulates PI3K by removing the phosphate from its Ser608-p85 residue, which is autophosphorylated by the catalytic subunit of this enzyme [62, 63]. Thus, it was first reported that a tightly associated protein Ser/Thr kinase phosphorylates the p85 subunit of the PI3K and causes its inactivation upon stimulation with middle T antigen [63]. It was later shown that this associated kinase is in fact the 110 kDa catalytic subunit of PI3K. Thus, the catalytic subunit can act as a dual specificity lipid and protein kinase. By phosphorylating the Ser608-p85, it inactivates both the lipid and protein kinase activities of this enzyme [49, 62]. This autophosphorylation is induced by various stimuli, suggesting that it may act as a negative feedback of the PI3K pathway causing its inactivation. Importantly, it was shown that this phosphorylation is counteracted by PP2A [62, 63], which consequently permits PI3K activation. However, the components and regulation of PP2A in this system have not been determined up to now. Here we show that this regulation might be mediated by an IGBP1-containing PP2A. Furthermore, we show that the regulation by PP2A also occurs in non-stimulated cells to contribute to a relatively high basal activity of the lipid kinase. In our system, stimulation disrupted the interaction between PP2A and PI3K, leading to inactivation of the lipid kinase activity. This seems a unique event that is unlikely to occur upon survival/growth stimulations, which have effects opposite to the one described here.

PP2A is one of the most important regulators of the protein Ser/Thr kinase AKT, as shown previously [25]. The dephosphorylation of this kinase results in its inactivation, and consequently negatively regulates its downstream promotion of survival and/or metabolism related processes. PP2A heterotrimer consisting of PR56β is required for dephosphorylation of residues p-Ser473 and p-Thr308, at least upon insulin stimulation [27]. We therefore entertained the possibility that upon release from PI3K, the IGBP1 is replaced by PR56β to induce the inactivating dephosphorylation of AKT. No effect of the SiRNA directed to PR56β was detected either in our initial screen (Fig. 4), or when we knocked down this isoform. In addition, no change in the interaction of AKT with PR56β was detected in αT3-1 and PC3 cells. Thus, IGBP1 likely provides PP2A with the ability to recognize AKT, at least in our conditions. As mentioned above, IGBP1 might be the main regulator of the PI3K-AKT-mTOR pathway, at least when downregulated upon GqPCR stimulation.

## Conclusions

We have previously found that, in certain cells, activation of GqPCRs results in AKT dephosphorylation and inactivation that leads to JNK activation and apoptosis [48]. Here we deciphered the molecular mechanism responsible for this pathway. We found that the main regulator of the effect is PP2A, which switches its interaction from PI3K to AKT upon GqPCR stimulation (“PP2A switch”). In resting cells, a heterodimer of PP2A, composed of IGBP1 and PP2Ac, interacts with PI3K to dephosphorylate its inhibitory Ser608-p85, and consequently induce high basal PI3K-AKT activity. Upon stimulation, PP2A detaches from PI3K, and subsequently binds as a holoenzyme of PP2Ac, IGBP1 and PP2Aa to AKT to induce its dephosphorylation. This process results in the inactivation of the two kinases and therefore allows activation of the JNK cascade leading to apoptosis. While PP2Ac and IGBP1 are essential for this action, PP2Aa binding does not play a role in the AKT-inactivating switch. The fact that PP2Aa and IGBP1 can simultaneously associate with PP2Ac only upon stimulation resolves the controversy regarding this issue, as the stimulation probably results in a conformational change that allow the simultaneous binding. In addition, it shows for the first time that the complex formation of PP2A can be modulated by extracellular stimulation. Thus, our results delineate a mechanism that involves PI3K and AKT that induces a death-receptors independent apoptosis in various systems.

## Supplementary Information

**Additional file 1:** Supplementary Figures (Figures S1–S5), Table (Table S1) and Full Blots.

## Data Availability

All data and materials are described in the text.

## Abbreviations

GnRH: Gonadotropin-releasing hormone
GPCR: G protein coupled receptors
IGBP: Immunoglobulin binding protein
PGF2α: Prostaglandin F2α
PI3K: Phosphoinositol-3 kinase
PKC: Protein kinase C
PP2A: Protein phosphatase 2A
