# Supplementary material for: GqPCR-stimulated dephosphorylation of AKT is induced by an IGBP1-mediated PP2A switch
Source: Cell Commun Signal. 2022 Jan 8;20:5. doi: 10.1186/s12964-021-00805-z (PMC8742922; doi:10.1186/s12964-021-00805-z)
Supplement: Supplementary file 2 — Additional file 1: Supplementary Figures (Figures S1–S5), Table (Table S1) and Full Blots. [file 12964_2021_805_MOESM1_ESM.pdf]

Supplementary Figures, Table and Full Blots

GqPCR-stimulated dephosphorylation of AKT is induced by an IGBP1-mediated PP2A switch

Guy Nadel, Zhong Yao, Ehud Wainstein, Izel Cohen, Ido Ben Ami, Amir Schajnovitz, Galia Maik-Rachline, Zvi Naor, Benjamin A. Horwitz, and Rony Seger

Figures

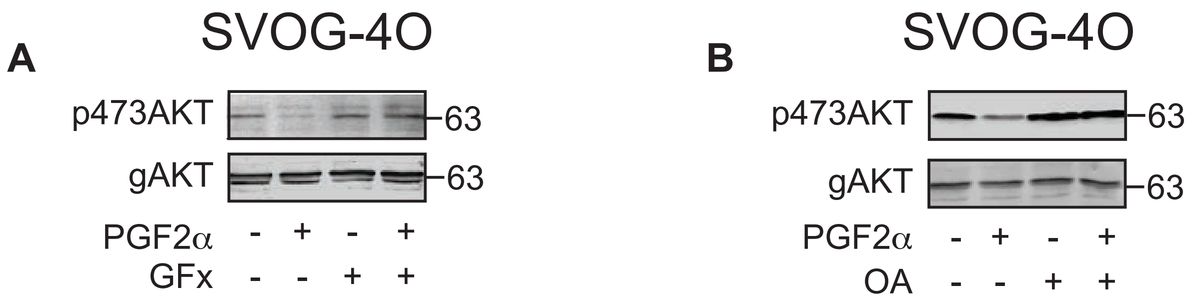

Figure S1. Reduced AKT phosphorylation induced by PGF2α is mediated by PKC and PP2A. (A) AKT dephosphorylation is mediated by PKC. Serum-starved SVOG-4O cells were pretreated with GFx, or left untreated. Then the cells were stimulated with GnRH-a/PGF2α or DMSO control, and then harvested. Phosphorylation of AKT was determined using Western blotting with the indicated Abs. (B) AKT dephosphorylation is mediated by PP2A. Serum starved SVOG-4O cells were pretreated with okadaic acid (OA, 0.5 μM), or left untreated. Then the cells were stimulated with GnRH-a/PGF2α or DMSO control, and then harvested. Phosphorylation of AKT was determined using Western blotting with the indicated Abs.

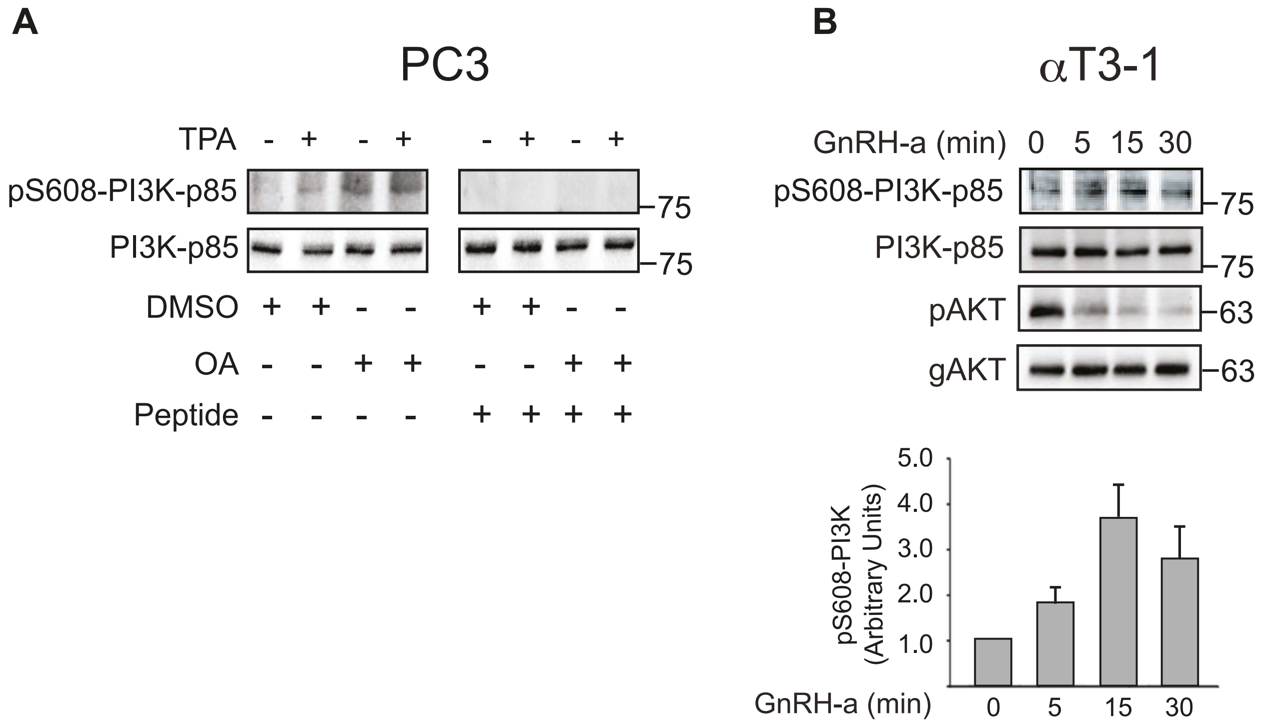

Figure. S2. **Characterization of the anti pSer608-p85 Ab.** (A) Specificity of the Ab. PC3 cells were grown to 70% confluence, starved for 16 hr and treated with OA (0.5 µM, 20 minutes) prior to stimulation with TPA (250nM, 30 min; +) or DMSO control (-). Then the cells were harvested and immunoblotted with the indicated Abs (left panel). In parallel, the immunogenic peptide used for generating the pSer608-p85 Ab (100 µg/ml) was incubated with the Ab (1 hr, 23^{0}C) and these treated Ab were used for the immunoblotting of the same extracts instead of the original one (right panel). (B) GnRH-a induces Ser608-p85 phosphorylation in αT3-1 cells. Serum starved αT3-1 cells were treated with GnRH-a (0.1 µM, indicated times), and then harvested. The extracts were then subjected to Western blotting with the indicated Abs. The bar-graphs in the bottom represents the average and standard error of two experiments.

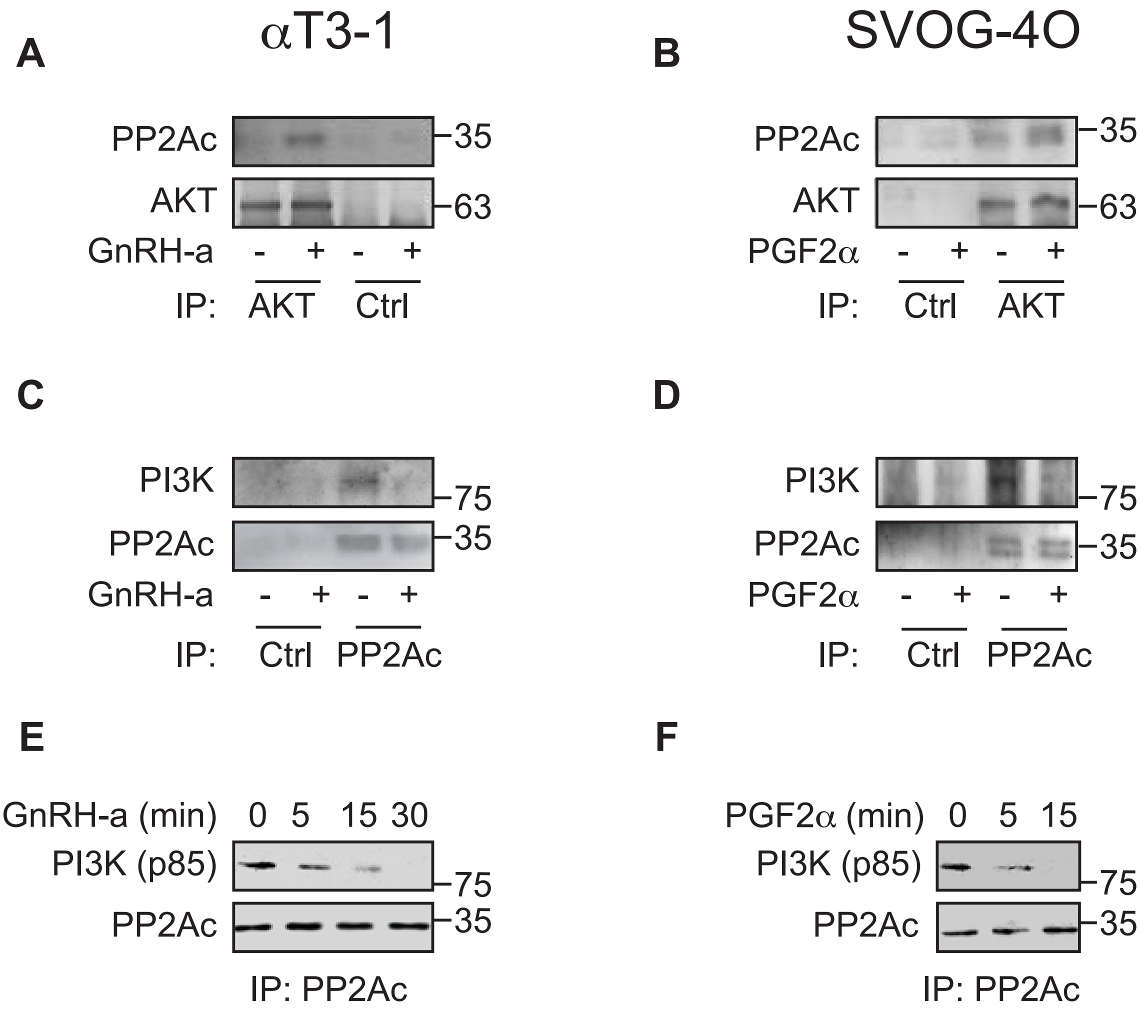

Figure S3. Modulation of PP2Ac interaction with AKT and PI3K upon PGF2α/GnRH-a stimulation. (A,B) Increased AKT interaction with PP2Ac upon GnRH-a/PGF2α treatment. Serum-starved αT3-1 (A) and SVOG-4O (B) were treated with GnRH-a (0.1 μM) or PGF2α (10 μM) for 30 min and then the cells were harvested. AKT was IPed with anti-AKT Ab, or IgG (control), and the IPed proteins as well as the CoIPed PP2Ac were detected by Western blotting with the indicated Abs. (C,D) Decreased PI3K interaction with PP2Ac upon GnRH-a/PGF2α treatment. Serum-starved αT3-1 (C) and SVOG-4O (D) were treated with GnRH-a (0.1 μM) or PGF2α (10 μM) for 30 min and then the cells were harvested. PP2Ac was IPed with its cognate Ab (or IgG control), and the CoIPed PI3K-p85 as well as the IPed PP2Ac were detected by Western blotting with the indicated Abs. (E,F) Time course of PI3K detachment from PP2Ac upon GnRH-a/PGF2α treatment. Serum-starved αT3-1 (E) and SVOG-4O (F) were treated with GnRH-a (0.1 μM) or PGF2α (10 μM) for the indicated times and then the cells were harvested, and PP2Ac was IPed with its Ab (or IgG control), and the IPed proteins as well as the CoIPed p85-PI3K were detected by the indicated Abs.

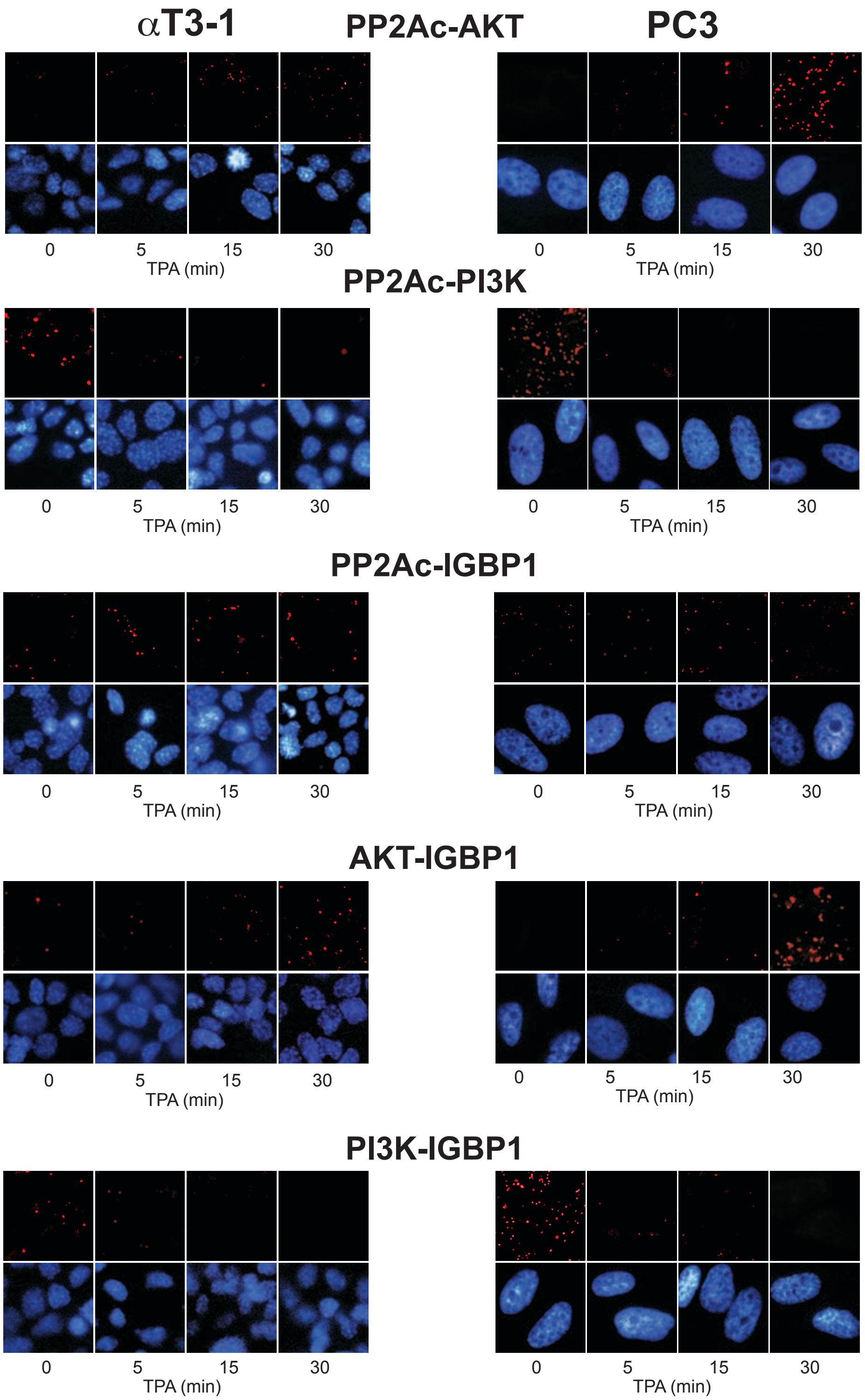

Figure S4. Monitoring PKC-dependent PP2Ac interactions with PI3K, AKT and IGBP1 by PLA. αT3 (left) and PC3 (right) cells were cultured on cover slips, followed by serum starvation and TPA stimulation (250 nM for the indicated times). Cells were fixed and the protein-protein interactions were detected using PLA kit with the indicated Abs (red). Nuclei were stained with DAPI (blue).

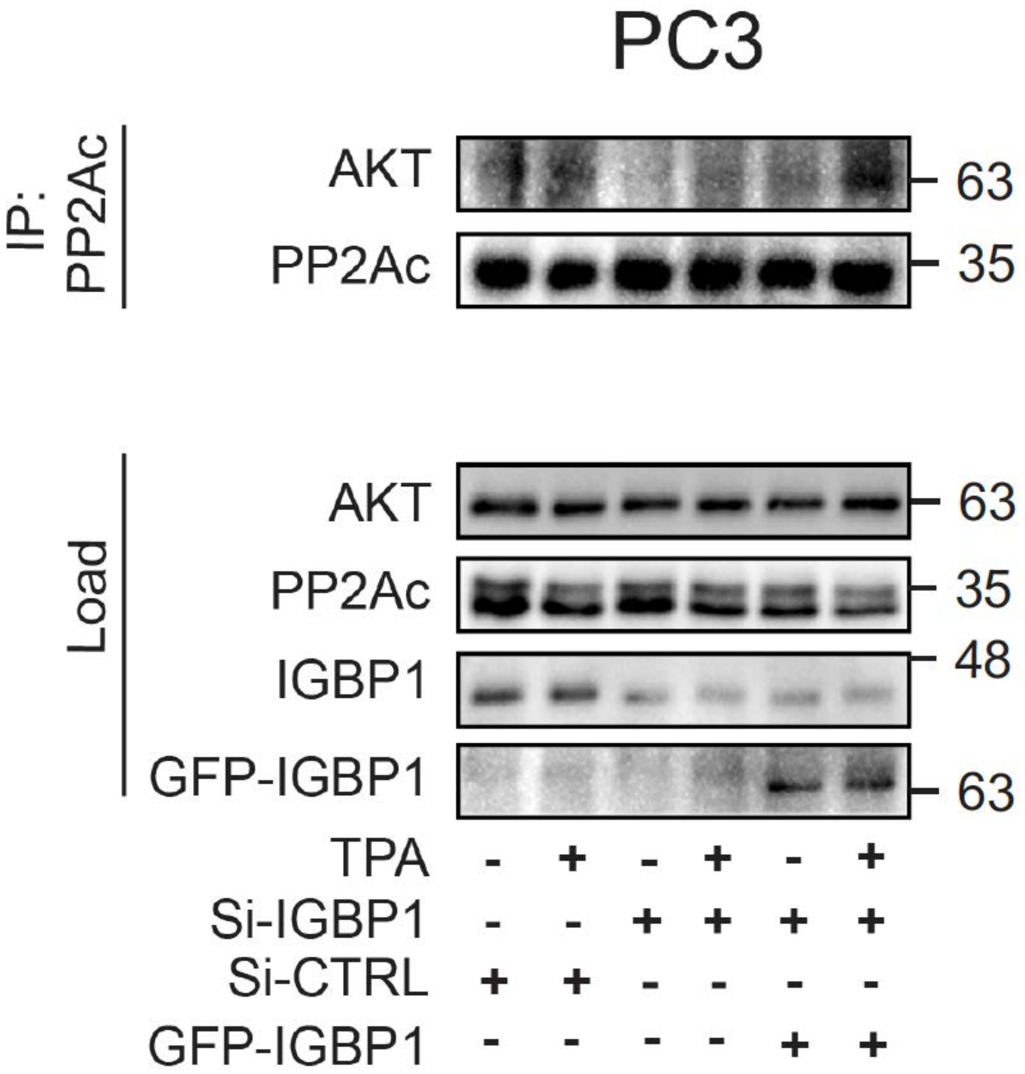

Figure S5. Confirming the on-target effect of the SiRNA of IGBP1. PC-3 cells were grown in 10% FCS and then transfected with IGBP1-GFP (lanes 5-6) or left untreated (lanes 1-4). Then, cells were transfected for 6 hr with siRNA of IGBP1 (50 nM; lanes 3-6) or siRNA control (50 nM, CTRL) (lanes 1-2). The SiRNA transfected medium was then replaced by starvation medium 0.1% FCS RPMI for additional 12 hr, after which the cells were either stimulated with TPA (250nM, 30 min, lanes 2,4,6; +) or left untreated (lanes 1,3,5; -). The cells were then harvested, PP2Ac was IPed from cell extracts using anti PP2Ac Ab, and the CoIPed AKT was detected using Western blotting with anti AKT. The initial amounts of extracts were probed with anti AKT, PP2Ac, IGBP1 and GFP Abs (Load).

Table

CoIP with:	IGBP1	AKT
PP2Aa	PPP2R1A (PP2Aa alpha isoform)	PPP2R1A (PP2Aa alpha isoform)
PP2Ab	PPP2R2A and PPP2R4	--
PP2Ac	PPP2CA	PPP2CA (weak)
Others	IGBP1	IGBP1

Table S1 – Mass-spectrometric analysis of the PP2A components that were CoIPed with IGBP1 and AKT from stimulated cells.

Full blots

Fig. 1A

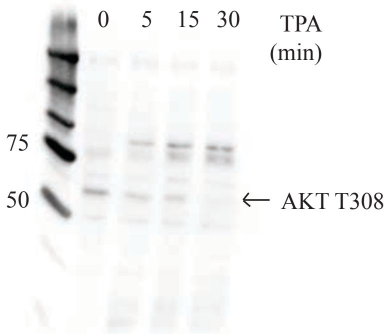

Figure 1A top blot

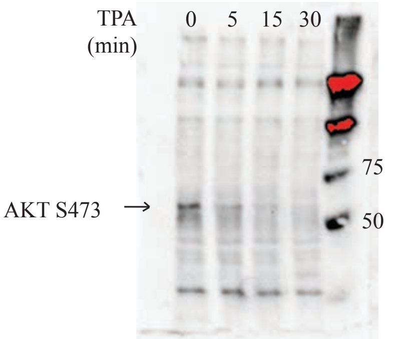

Figure 1A middle blot

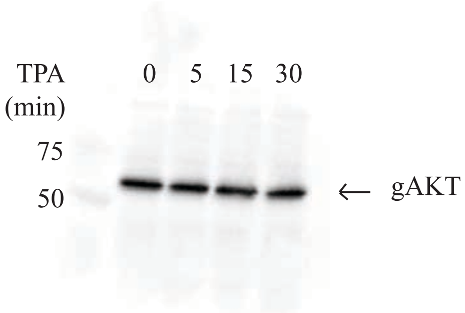

Figure 1A bottom blot

Fig. 2E

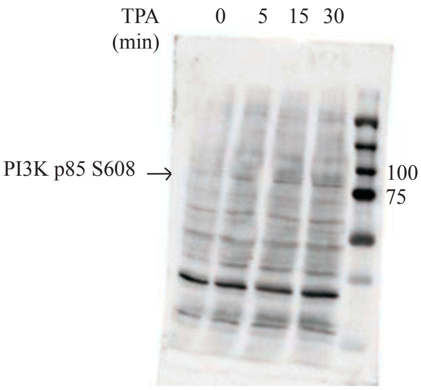

Figure 2E top blot

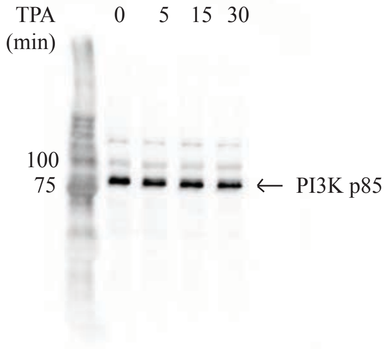

Figure 2E bottom blot

Fig. 3A

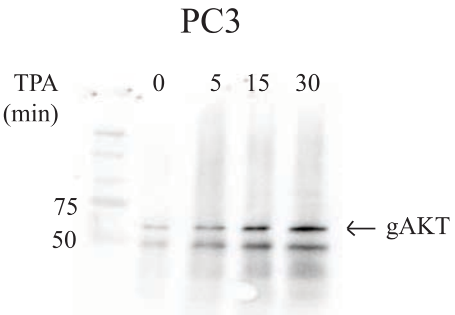

Figure 3A top blot (IP)

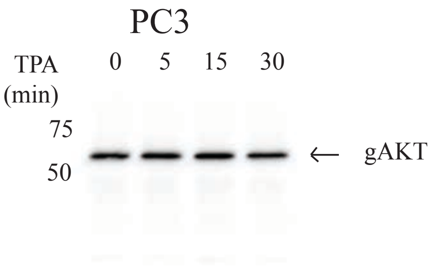

Figure 3A top blot (load)

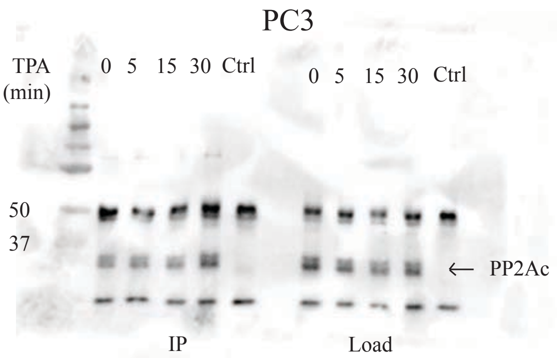

Figure 3A second(IP) and 4th (load) blots

Fig. 3D

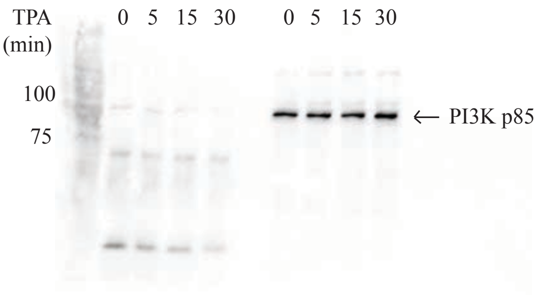

Figure 3C top (IP) and third (load) blots

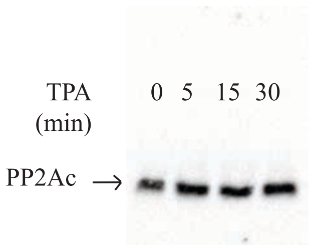

Figure 3C second blot (IP)

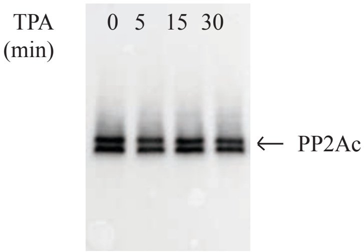

Figure 3C 4th blot (Load)

Fig. 4A

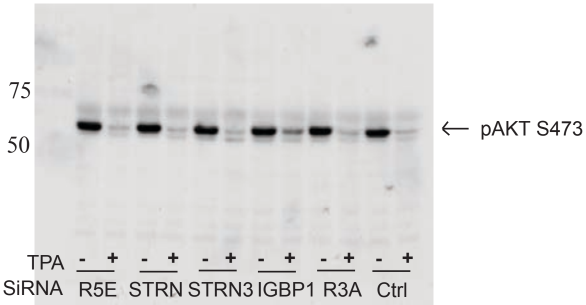

Figure 4A bottom panel bottomblot

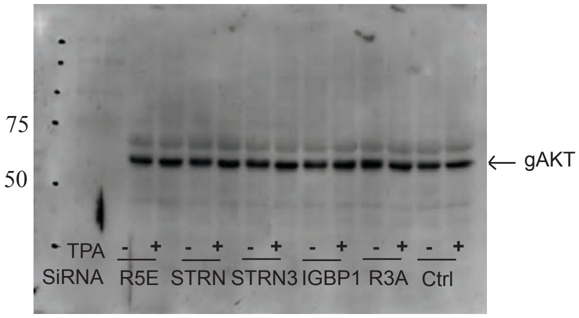

Figure 4A bottom panel top blot

Fig. 4F

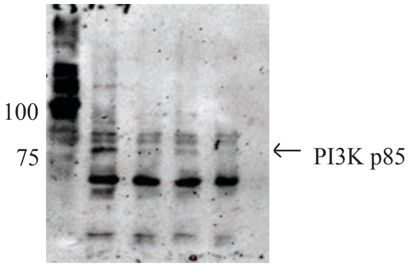

Figure 4F left panel top blot (IP)

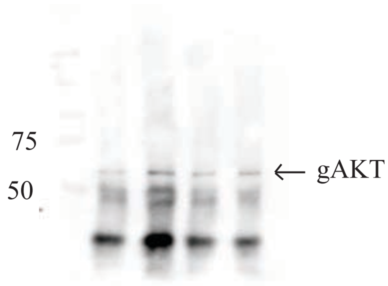

Figure 4F left panel second blot (IP)

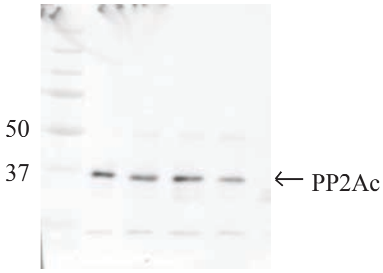

Figure 4F left panel third blot (IP)

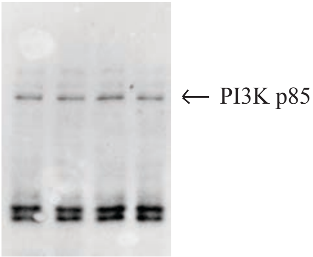

Figure 4F left panel 4th blot (load)

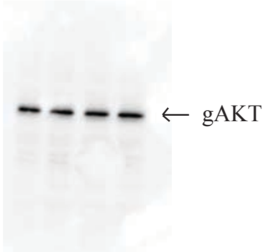

Figure 4F left panel 5th blot (load)

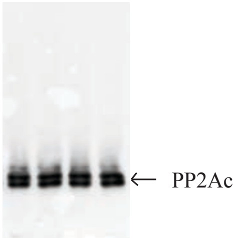

Figure 4F left panel 6th blot (load)

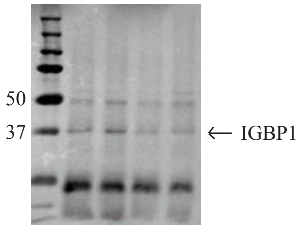

Figure 4F left panel 7th blot (load)

Fig. 5A

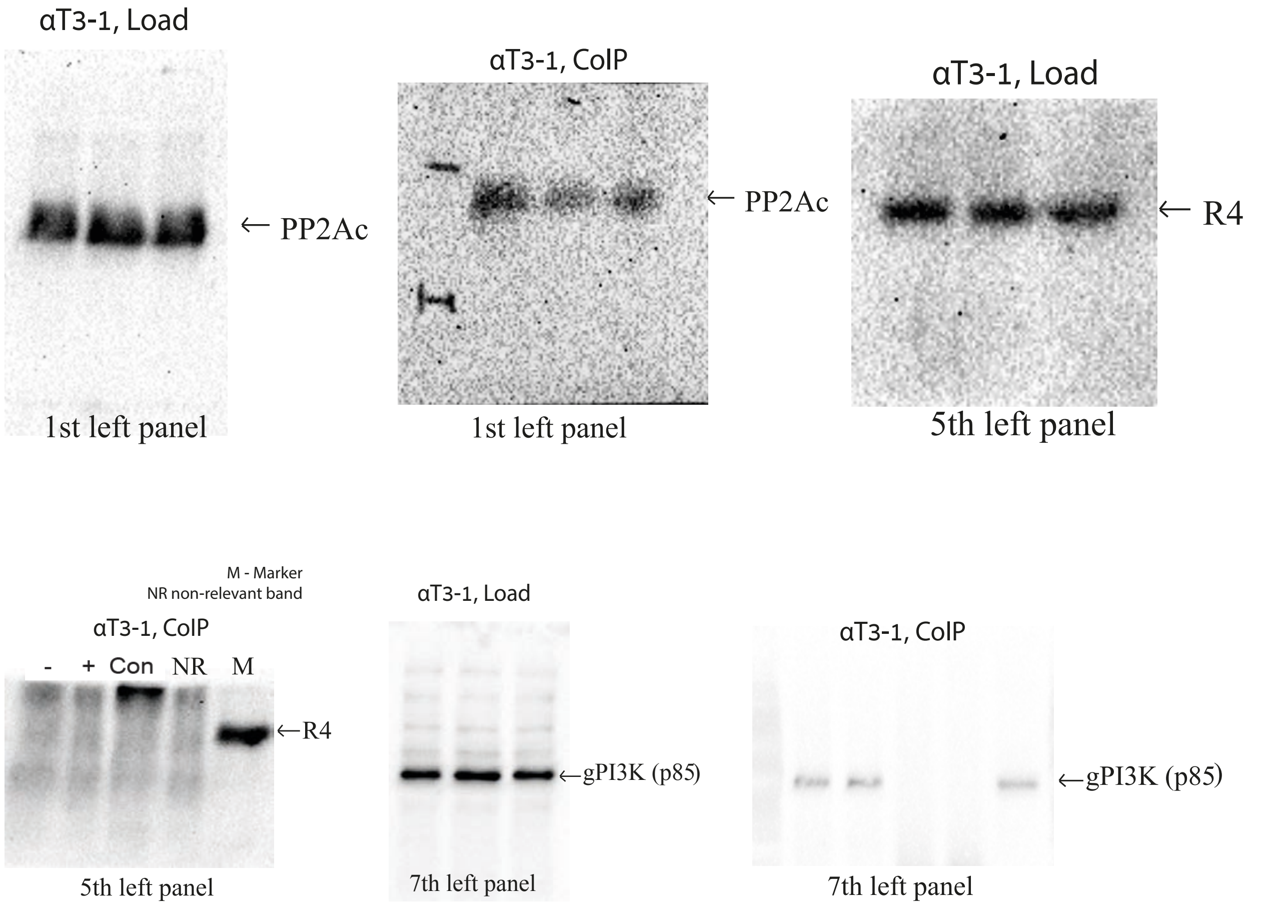
αT3-1, Load
← PP2Ac
1st left panel
αT3-1, CoIP
← PP2Ac
1st left panel
αT3-1, Load
← R4
5th left panel
M - Marker
NR non-relevant band
αT3-1, CoIP
- + Con NR M
←R4
5th left panel
αT3-1, Load
←gPI3K (p85)
7th left panel
αT3-1, CoIP
←gPI3K (p85)
7th left panel

Fig. 7B

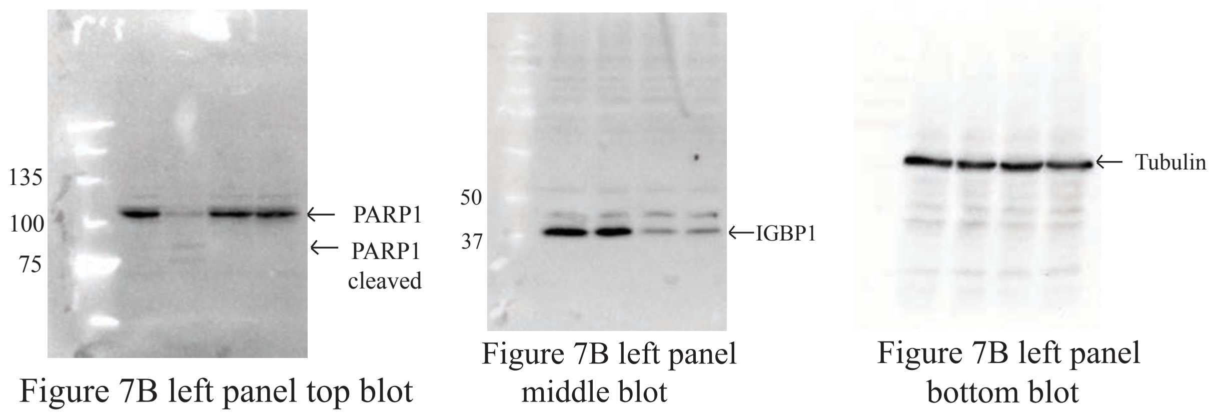
135
100
75
← PARP1
← PARP1 cleaved
Figure 7B left panel top blot
50
37
←IGBP1
Figure 7B left panel middle blot
← Tubulin
Figure 7B left panel bottom blot
